# PD137 Reviewing The Health Technology Assessment And Regulatory Policy Landscape On Acceptability Standards For Real-World Evidence - Initial Findings

**DOI:** 10.1017/S026646232400374X

**Published:** 2025-01-07

**Authors:** Katharine Cresswell, Ravinder Claire, Tuba Saygin Avsar, Thomas Hudson, Rose Purcell, Rita Peeters, Lotte Groth Jensen, Yun Chen, Stine Hasling Mogensen, Fabio Bisordi, Gracy Crane, Dalia Dawoud, Heather Colvin

## Abstract

**Introduction:**

There is an increasing number of policy and guidance documents on the use and acceptability of real-world evidence (RWE) to support regulatory and health technology assessment (HTA) decision-making. The Innovative Health Initiative Integration of Heterogeneous Data and Evidence towards Regulatory and HTA Acceptance (IDERHA) partnership is undertaking a global landscape review of these documents to understand where there is consensus and divergence, and where further policy development is needed.

**Methods:**

A literature search of the MEDLINE and Embase databases was performed, in addition to handsearching the websites of specific HTA and regulatory organizations. All policies, standards, frameworks, and guidance documents on requirements for acceptable RWE data use published from 2017 were included. Two reviewers independently extracted data using a standard data extraction form that was pilot tested before use. Any discrepancies between the reviewers were resolved by consensus. Extracted data are currently being analyzed by researchers with regulatory or HTA expertise. A workshop held in October 2023 sought input from experts on analysis plans.

**Results:**

The initial literature search yielded 3,184 results. After screening against the inclusion criteria, a total of 87 documents were selected for full-text review (21 HTA and 62 regulatory documents). Of these, 32 were identified as key documents and prioritized for initial review. Key themes in the documents, including transparency, data collection, study design, and data quality, were identified and validated in a workshop with five regulatory or HTA experts. Data extraction is ongoing for the remaining documents and any further themes identified will be added. Any gaps and areas of divergence will be identified, so they can be addressed by future IDERHA work.

**Conclusions:**

This review assessed the increasingly complex global landscape of regulatory and HTA policies and guidance on the use of RWE. Through the exploration of similarities, differences, and gaps in these policies, this work will extend the current understanding of best practice and identify areas that need development of further guidance.

